# An evaluation of errors in the mitochondrial COI sequences of Hydrachnidia (Acari, Parasitengona) in public databases

**DOI:** 10.1007/s10493-022-00703-0

**Published:** 2022-02-25

**Authors:** María L. Peláez, José L. Horreo, Ricardo García-Jiménez, Antonio G. Valdecasas

**Affiliations:** 1 Museo Nacional de Ciencias Naturales, C/José Gutiérrez Abascal, 2, 28006 Madrid, Spain; 2 UMIB Research Unit of Biodiversity (UO, CSIC, PA), Oviedo University - Campus Mieres, C/Gonzalo Gutiérrez Quirós s/n, 33600 Mieres, Spain; 3 Department of Genetics, Physiology and Microbiology, Complutense University of Madrid, C/Jose Antonio Novais 12, 28040 Madrid, Spain

**Keywords:** BOLD, Cryptic diversity, GenBank, Phylogeny, Species identification, Water mites

## Abstract

Public molecular databases are fundamental tools for modern taxonomic studies whose usefulness rely on the soundness of the data within them. Here, we study potential errors that can arise along the data pipeline from sampling, specimen identification and molecular processing (digestion, amplification and sequencing) to the submission of sequences to these databases by using the DNA sequences of Hydrachnidia (Acari, Parasitengona) as a case study. Our results indicate that molecular information is available for only about 3% of the Hydrachnidia species known to date; yet, within this small percentage, errors are present in almost 5% of the species analyzed (0.5% of the sequences and almost 11% of the genera). This study underscores the scarcity of genetic data available for Hydrachnidia, but also that the proportion of errors in DNA sequences is relatively small. Even so, it highlights the danger associated with using DNA sequences from public databases, particularly for species identification, and reinforces the need for greater quality control measures and/or protocols to avoid an intensification of errors in the (post) genomics era. Finally, our study emphasizes that potential errors may also reveal cryptic diversity within a species.

**Supplementary Information:**

The online version contains supplementary material available at 10.1007/s10493-022-00703-0.

## Introduction

The quality of empirical data is the basis for hypothesis testing, model building and theory generation (Glass 2007). In the field of taxonomy, robust data based on morphology, behaviour or any other characters that may facilitate the discovery and/or identification of taxa should be taken into account for species resolution (e.g., Alarcon-Elbal et al. 2020). The increasing use of molecular data, an additional powerful resource for characters, is leading the field towards being an exact science (Page et al. 2005). Although DNA barcoding (Hebert et al. 2003), which uses DNA fragments as a means to identify species, is nowadays a common tool, we are still far from that presumptive future (Janssen et al. 2017). The use of erroneous DNA sequences that are stored in public databases could negatively impact research. For example, we can infer erroneous phylogenies and phylogeographic patterns, obtain inaccurate genetic variability estimations or even misidentify the actual species of a specimen if its identification is based on a molecular comparison (e.g., BLAST). Subsequently, all interpretations based on such analyses could be wrong.

As with other types of characters, molecular sequences are prone to various kinds of potential errors, with the following being among the more common: (a) laboratory mismanagement of samples (including DNA contamination) that leads to the incorrect assignment of a particular sequence to another taxon; (b) incorrect identification of organisms; (c) inadequate molecular marker selection; and (d) errors during sequence submission to databases. Other less frequent errors also occur, such as using a generic abbreviation that may cause confusion between two taxa with the same specific name (e.g., *Hydrachna crassipalpis* and *Hydryphantes crassipalpis*).

Whereas minor errors can typically be easily found and corrected, others may pass undetected, which could lead to further mistakes, as has been described for other sequences such as in viruses (Wagner and Bodem 2017) and fishes (Li et al. 2018). Moreover, problematic DNA sequences, as a result of taxonomic problems, errors in identification or genetic introgression, among others, have been found in public databases (Harris 2003; Lis et al. 2016), leading to doubts about the reliability of such resources. Anomalous patterns in DNA barcode data may also be indicative of cryptic species, morphologically identical species that have developed reproductive barriers among them (e.g., Bickford et al. 2007), which are widely known to occur in Acari (Scoracka et al. 2015). In this way, DNA sequences deemed to be problematic or erroneous may actually be a signal of cryptic diversity. Genetic introgression or hybridization may also lead to anomalies, as mitochondrial information may identify the maternal species of a hybrid though the morphology may be associated with that of the paternal species (Pelaez et al. 2018). Incomplete lineage sorting, which can cause discordance in gene trees and, therefore, lead to incorrect inferences of phylogenetic relationships among species (Linder and Rieseberg 2004), could also give rise to a misidentification if the phylogenetic tree is used to search for potential DNA database conflicts.

Potential incongruences that may arise from the increasing use of molecular data in taxonomic studies, such as those outlined above, have been little explored for the highly diverse Hydrachnidia (water mites) clade, for which species identification can be challenging. This clade is the third largest group of animals inhabiting freshwater habitats in terms of number of species: approximately 7000 species distributed in 439 genera are known worldwide. They inhabit all types of habitats, except those located above the permanent snow line (Cook 1974). Many Hydrachnidia species are parasitic and use freshwater insects at the larval, nymph and adult stages as hosts. They are also predators of insects and crustaceans and, thus, play an important role in freshwater aquatic ecosystems (Proctor et al. 2015).

As a case study, we assess the potential level of error associated with water mite sequences from GenBank and discuss the possible sources of these errors. Although the extent of errors for Hydrachnidia sequences available in either GenBank or the Barcode of Life Data (BOLD) system is unknown, given the relatively low sequence coverage for the group, the impact of any error may prove significant for future molecular studies. For our analyses, we assessed and compared sequences of the cytochrome oxidase subunit I (COI) gene as it is, to date, the most widely available marker for the clade (see below). Although, in our study, we are not directly concerned with the more general problem of the resolving power of DNA barcoding for species identification and discovery (Meyer and Paulay 2005), our findings may provide additional reasons to caution the utility of barcoding for such purposes.

## Materials and methods

A search for ‘Hydrachnidia’ was performed in GenBank (https://www.ncbi.nlm.nih.gov/genbank) on 24 July 2019 in order to determine the highest possible number of specific genetic sequences available in the database for the group. A total of 5432 sequences was found, of which 4914 were of COI.

Sequences were aligned (Supplementary Material S1) using the MAFFT online server (https://mafft.cbrc.jp/alignment/server/). The progressive method FFT-NS-1 was used because of the high number of sequences analyzed (as recommended for more than 2000 sequences). With this alignment, a maximum likelihood (ML) phylogenetic tree was reconstructed using IQ-TREE (Nguyen et al. 2015) on its online server (http://iqtree.cibiv.univie.ac.at) and the SH-aLRT branch test, as recommended for analyses with a high number of sequences (Minh et al. 2013), with 1000 replicates (as recommended by Guindon et al. 2010). A *Leptus* sp. COI sequence (accession number HM379322) was used as the outgroup in this analysis. The tree was visualized in FigTree (http://tree.bio.ed.ac.uk/software/figtree/), and nodes with bootstrap values >80 were considered as supported (Minh et al. 2013).

Potential erroneous sequences and/or species identifications were searched for in the tree by comparing the phylogenetic position of individuals attributed to the same species, and of species attributed to the same genus. To do this, we examined each sequence in the phylogenetic tree and compared it with the phylogenetic positions of the other sequences of the same species.

In addition, we cross-referenced GenBank sequences with BOLD (http://www.boldsystems.org) to determine the extent to which sequences are similarly identified in the two databases. This verification included the species-specific sequences downloaded from GenBank and then submitted to BOLD on 17 October 2019. As BOLD requires the forward strand sequence for submission, the few reverse strand sequences found in GenBank were transformed to forward ones in Reverse Complement (https://www.bioinformatics.org/sms/rev_comp.html) and submitted again for identification.

## Results

Only 56 Hydrachnidia genera, accounting for approximately 13% of known ones, and 203 species are represented in the 4914 COI sequences downloaded from GenBank (Tables 1 and 2). Of these, 13 were excluded from further analyses as they represented a non-existent species, Hydracarina or nonstandard taxonomic categories. A similar analysis could not be done in BOLD because many of the sequences are ‘private’ and thus not available for download.

**Table 1:** A summary of the 4901 COI molecular sequences from GenBank that were included in this study

Taxa	Family	Genera	Species	Total
Hydrovolziidae	0	1	1	2
Limnocharidae	1	7	1	9
Eylaidae	0	43	0	43
Hydrachnidae	0	29	2	31
Hydrodromidae	14	44	3	61
Hydryphantidae	59 (+ 3)	73	3	138
Thermacaridae	0	0	1	1
Anisitsiellidae	18	0	0	18
Lebertiidae	41	303	32	376
Sperchontidae	113	105	19	237
Torrenticolidae	5	43	621	669
Teutonidae	0	1	1	2
Oxidae	2	25	2	29
Aturidae	6	26	1	33
Feltriidae	0	1	0	1
Hygrobatidae	171	50	153	374
Pontarachnidae	0	0	1	1
Wettinidae	0	1		1
Limnesiidae	58 (+ 13)	202	19	292
Pionidae	356	453	70	879
Unionicolidae	142	363	75	580
Arrenuridae	108	748	192	1048
Bogatidae	0	0	1	1
Mideopsidae	13	31	2	46
Krendowskiidae	12	8	2	22
Laversiidae	0	4	0	4
Mideidae	0	2	0	2
Neoacaridae	1	0	0	1
Total	1120 (+ 16)	2563	1202	4901

The list, sorted by taxonomic category, indicates the number of sequences identified to the species level or to only the genera or the family level. A total of 4914 sequences were downloaded from GenBank; however, one sequence belonged to a nonexistent *F. thyasidae*, four to Hydracarina and the other eight to nonstandard categories in the taxonomy of water mites. Numbers in parentheses indicate sequences that were assigned to the subfamily rank. The total number of sequences with vouchers was 1775

**Table 2:** Species list and the number of Hydrachnidia COI sequences (with more than 500 nucleotides) found in GenBank for each species

Genus	Species	Sequences	Genus	Species	Sequences
*Arrenurus*	*affinis*	1	*Testudacarus*	*americanus*	4
	*albator*	1		*dawkinsi*	7
	*americanus*	14		*deceptivus*	2
	*apetiolatus*	7		*dennetti*	10
	*bicuspidator*	1		*elongatus*	6
	*birgei*	1		*harrisi*	16
	*biscissus*	1		*hitchensi*	13
	*bleptopetiolatus*	3		*hyporhynchus*	3
	*bruzelii*	2		*kirkwoodae*	2
	*cardiacus*	1		*minimus*	29
	*cheboyganensis*	3		*oblongatus*	8
	*compactus*	2		*rectangulatus*	1
	*crassicaudatus*	1		*rollerae*	3
	*crenellatus*	4		*smithi*	3
	*cuspidifer*	1		*vulgaris*	32
	*cylindratus*	1	*Teutonia*	*cometes*	1
	*drepanophorus*	3	*Thermacarus*	*nevadensis*	6
	*fimbriatus*	1	*Torrenticola*	*amplexa*	2
	*fissicornis*	4		*biscutella*	3
	*globator*	1		*bondi*	1
	*hungerfordi*	1		*caerulea*	2
	*inexploratus*	1		*copipalpa*	10
	*intermedius*	4		*delicatexa*	13
	*longicaudatus*	6		*dunni*	10
	*lyriger*	1		*ellipsoidalis*	24
	*magnicaudatus*	1		*elongata*	2
	*major*	1		*elusiva*	1
	*manubriator*	6		*erectirostra*	4
	*marshallae*	11		*flangipalpa*	6
	*maryellenae*	1		*glomerabilis*	4
	*mediorotundatus*	1		*gnoma*	5
	*megalurus*	1		*gorti*	7
	*mucronatus*	1		*hoosieri*	1
	*neumani*	3		*intiriorensis*	4
	*perforatus*	1		*irapalpa*	15
	*planus*	12		*karambita*	2
	*pustulator*	1		*larvata*	2
	*reflexus*	7		*longitibia*	1
	*robustus*	1		*lukai*	2
	*securiformis*	3		*lundbladi*	3
	*setiger*	2		*magnexa*	14
	*sinuator*	3		*malarkeyorum*	8
	*solifer*	9		*manni*	3
	*stecki*	2		*mjolniri*	12
*Arrenurus*	*suecicus*	1	*Torrenticola*	*mulleni*	10
	*tricuspidator*	2		*multiforma*	38
	*truncatellus*	1		*neoanomala*	10
	*wardi*	53		*nigroalba*	10
*Atractides*	*cognatus*	1		*nortoni*	12
	*latisetus*	1		*olliei*	1
	*propatulus*	1		*pacificensis*	8
*Aturus*	*scaber*	1		*pearsoni*	4
*Australotiphys*	*barmutai*	1		*pendula*	2
*Coaustraliobates*	*cortipes*	1		*pollani*	6
*Debsacarus*	*oribatoides*	6		*projector*	7
*Horreolanus*	*orphanus*	1		*racupalpa*	1
*Hydrachna*	*conjecta*	1		*rala*	1
	*globosa*	1		*raptor*	20
*Hydrodroma*	*torrenticola*	1		*raptoroides*	3
*Hydrovolzia*	*placophora*	1		*regalis*	1
*Hydryphantes*	*waynensis*	1		*robisoni*	1
*Hygrobates*	*fluviatilis*	76		*rockyensis*	7
	*foreli*	2		*sellersorum*	15
	*hamatus*	2		*sharkeyi*	5
	*longipalpis*	1		*shubini*	5
	*marezaensis*	5		*sierrensis*	33
	*nigromaculatus*	44		*skvarlai*	2
	*norvegicus*	1		*solisorta*	10
	*persicus*	1		*tahoei*	25
	*trigonicus*	2		*tricolor*	9
	*turcicus*	15		*trimaculata*	27
*Krendowskia*	*similis*	2		*tysoni*	10
*Lebertia*	*inaequalis*	7		*ululata*	2
	*madericola*	16		*unimaculata*	8
	*maderigena*	3		*ventura*	5
	*porosa*	2		*walteri*	14
	*quinquemaculosa*	4		*welbourni*	1
*Limnesia*	*marshallae*	1	*Unionicola*	*abnormipes*	1
	*undulatoides*	16		*aculeata*	1
*Limnochares*	*americana*	1		*agilex*	3
*Litarachna*	*communis*	1		*amandita*	1
*Mideopsis*	*roztoczensis*	1		*arcuata*	7
*Oxus*	*nodigerus*	2		*chelata*	3
*Partnunia*	*steinmanni*	1		*crassipes*	17
*Piona*	*alpicola*	9		*dimocki*	2
	*coccinea*	7		*foili*	6
	*dispersa*	8		*formosa*	3
	*exilis*	2		*fulleri*	1
*Piona*	*imminuta*	4	*Unionicola*	*gailae*	1
	*longipalpis*	10		*hoesei*	1
	*pusilla*	13		*ischyropalpus*	1
				*kavanaghi*	1
	*stjordalensis*	9		*minor*	11
	*variabilis*	9		*parkeri*	4
*Protzia*	*squamosa*	1		*serrata*	2
*Sperchon*	*fuxiensis*	1		*smithae*	1
	*glandulosus*	3		*tumida*	1
	*plumifer*	8		*tupara*	1
	*rostratus*	4		*vamana*	1
	*violaceus*	1		*vikitra*	1
*Sperchonopsis*	*ecphyma*	1		*ypsilophora*	4
	*phreaticus*	1			

Just 24.5% of the sequences (1202) were identified to the species level, whereas 52.2% (2563 sequences) were identified to only the genus level, and 23.2% (1136 sequences) to subfamily and family level. The mean number of sequences per species was 5.87 (range 1–76; SD 9.07), and 128 of the species had >1 sequence (1125 sequences in total from species with more than one sequence). These sequences were used to compare the phylogenetic location of those of the same species in the inferred tree (which was constructed with all 4914 sequences). Cases in which a species with more than one sequence grouped with another species that only had a single sequence were not considered as errors. The genera were represented by an average of 6.83 sequences (range 1–60, SD 13.93). The mean number of sequences per genus was 67.46 (range 1–939; SD 165.74). Of the 56 genera, 23 had >10 sequences.

**Fig. 1:**
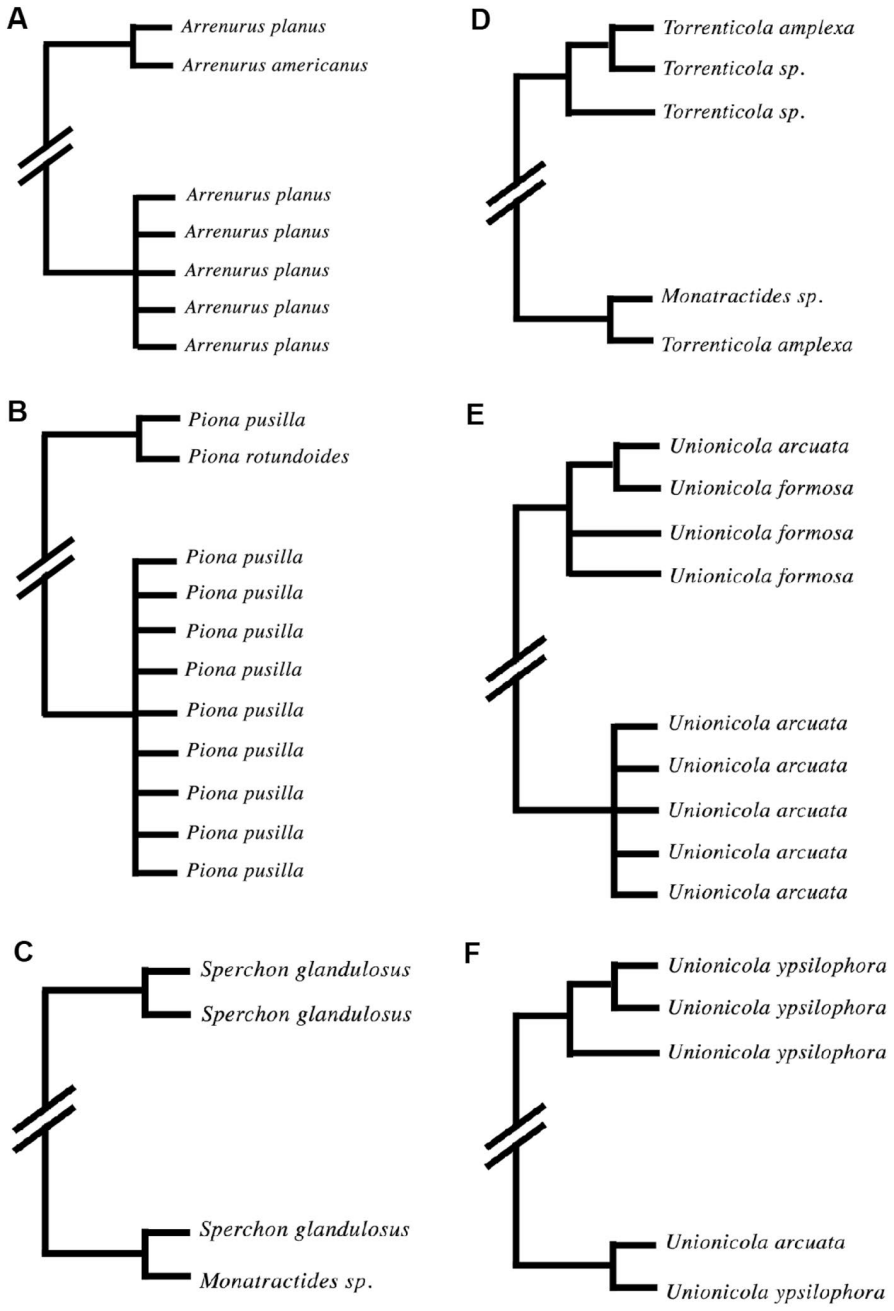
Schematic representation of the areas in the phylogenetic tree with problematic DNA sequences: **a** *Arrenurus planus*, **b** *Piona pusilla*, **c** *Sperchon glandulosus*, **d** *Torrenticola amplexa*, **e** *Unionicola arcuata* and **f** *U. ypsilophora*. ‘//’ indicates that several different species and clades are located between the represented sequences/clades. Branch lengths are not informative and for schematic purposes only. Sequences in polytomies are not necessarily 100% similar

The COI alignment of the 128 species was 672 bp long, which is considered sufficiently informative to reconstruct a phylogeny that likely reflects the species tree (Horreo 2012). However, in the obtained phylogenetic tree (Supplementary Material S2), six sequences (0.5% of the multiple sequences) belonging to six species (4.7% of the species with >1 COI sequence) from five genera (10.7% of the genera) did not resolve to their expected phylogenetic locations, suggesting an error in one sequence of each of the following six species: *Arrenurus planus*, *Piona pusilla*, *Sperchon glandulosus*, *Torrenticola amplexa*, *Unionicola arcuata* and *U. ypsilophora*. In four of the six cases (Fig. 1; Table 3), the sequence grouped with those belonging to another species within the same genus (*A. planus*, *P. pusilla*, *U. arcuata* and *U. ypsilophora*). In the other two cases, the sequences identified as *Torrenticola amplexa* and *Sperchon glandulosus* both grouped with those belonging to the genus *Monatractides*.

**Table 3:** Species whose sequences did not group as expected with others of the putative species, and the group to which each sequence likely belongs according to the phylogenetic analysis of COI sequences

Species	Group
*Arrenurus planus*	*Arrenurus americanus*
*Piona pusilla*	*Piona rotundoides*
*Sperchon glandulosus*	*Monatractides* sp.
*Torrenticola amplexa*	*Monatractides* sp.
*Unionicola arcuata*	*Unionicola formosa*
*Unionicola ypsilophora*	*Unionicola arcuata*

Of the 1202 sequences that were identified to the species level in GenBank, 649 (53.4%) corresponded to the same species identification in BOLD, although sequence similarity was not 100% in all cases. In the coincident sequences, the mean percentage of similarity was 99.76% (SD 0.46), and the range was between 97.34 and 100% (382 or 58.86% of the sequences showed 100% similarity). Ten of the GenBank sequences identified to the species level (0.84%) presented a high level of sequence similarity with a different species in BOLD (none of these corresponded to the sequences identified as erroneous in the phylogenetic tree comparison; see Table 3). The remaining 539 sequences do not share similarity with any public BOLD sequences.

For the GenBank sequences specified to at least genus level (1202 + 2563 = 3765 sequences), most were similarly identified in BOLD (90.34%). The remaining sequences (9.66%) corresponded to a different species identity in BOLD with a mean similarity of 99.46% (SD 0.65), and a range between 97.62 and 100% (similarity was 100% for the 29.25% of these sequences), indicating that species-level identification for Hydrachnidia is greater in BOLD than in GenBank.

Taxonomic discordance (due to potentially misidentified taxa) observed between GenBank and BOLD sequences that showed > 99% similarity and other discrepancies that arose in the comparison of the two databases are shown in Table 4. Even species for which a relatively good amount of data is known (e.g., have vouchers, images, publications) presented discordance. For example, two GenBank sequences of *Hydryphantes armentarius* paired with three *H. parmulatus* sequences in BOLD. Another minor disagreement concerned the reverse condition of a few of the *Unionicola* sequences from GenBank.

**Table 4:** Taxonomic discordance revealed by cross-referencing of sequences between GenBank and BOLD databases

GenBank code	Species in GenBank	% similarity	Voucher	Species in BOLD
KP836172	*Arrenurus affinis*	100	Yes	*A. neumani*
KP836172	*Arrenurus affinis*	99.81	Yes	*A. neumani*
KP836172	*Arrenurus affinis*	99.84	Yes	*A. compactus*
MG310481	*Arrenurus cheboyganensis*	99.63	Yes	*A. setiger*
MG317436	*Arrenurus cheboyganensis*	99.41	Yes	*A. setiger*
KP836179	*Arrenurus compactus*	100	Yes	*A. neumani*
KP836179	*Arrenurus compactus*	99.63	Yes	*A. neumani*
KP836179	*Arrenurus compactus*	99.44	Yes	*A. neumani*
KP836179	*Arrenurus compactus*	99.44	Yes	*A. affinis*
KP836179	*Arrenurus compactus*	99.24	Yes	*A. neumani*
KP836180	*Arrenurus compactus*	100	Yes	*A. neumani*
KP836180	*Arrenurus compactus*	99.63	Yes	*A. neumani*
KP836180	*Arrenurus compactus*	99.44	Yes	*A. neumani*
KP836180	*Arrenurus compactus*	99.44	Yes	*A. affinis*
KP836180	*Arrenurus compactus*	99.24	Yes	*A. neumani*
KP836225	*Arrenurus crassicaudatus*	99.25	Yes	*A. latus*
MG313303	*Arrenurus drepanophorus*	100	Yes	*A. mucronatus*
MG313501	*Arrenurus drepanophorus*	100	Yes	*A. mucronatus*
KP836207	*Arrenurus globator*	99.62–100	Yes	*A. tubulator*
KP836207	*Arrenurus globator*	99.06	Yes	*A. albator*
KP836192	*Arrenurus neumani*	99.63	Yes	*A. bicuspidator*
KP836192	*Arrenurus neumani*	99.06	Yes	*A. radiatus*
KP836236	*Arrenurus setiger*	99.81	Yes	*A. crenellatus*
EF633505	*Atractides latisetus*		No	–
JN018103	*Hydrachna conjecta*	99.02	Yes	*H. cruenta*
KY609985	*Hygrobates persicus*	99.07–99.22	Yes	*H. fluviatilis*
JN034739	*Piona dispersa*	99.34	Yes	*P. imminuta*
MN548141	*Hydryphantes armentarius*	99.54	Yes	*H. parmulatus*
MN548142	*Hydryphantes armentarius*	99.54	Yes	*H. parmulatus*
FJ218010	*Unionicola agilex*	*reversed*	No	–
FJ218014	*Unionicola agilex*	*reversed*	No	–
FJ218012	*Unionicola agilex*	53.42	No	Decapoda
GU550951	*Unionicola amandita*	82.64	No	*Sperchonopsis verrucosa*
FJ218006	*Unionicola chelata*	*reversed*	No	–
FJ218009	*Unionicola chelata*	53.57	No	Hymenoptera
FJ218018	*Unionicola chelata*	52.14	No	Hymenoptera
FJ524382	*Unionicola crassipes*	52.86	No	Psocodea
GU550954	*Unionicola fulleri*	85.39	No	Lepidoptera
FJ218017	*Unionicola ischyropalpus*	57.72	No	Mesostygmata

GenBank FASTA sequences were submitted for identification to BOLD. The species name associated with the GenBank sequence and the similarity to and species name of the corresponding BOLD sequence are also indicated. The voucher column indicates whether a voucher is associated with the GenBank sequence

## Discussion

The relative difficulty of taxonomic identification frequently depends on the accessibility of good diagnostic keys and the availability of experts for difficult cases. Taxonomy as a professional activity is in decline, in what is known as the ‘taxonomic impediment’ (Ebach et al. 2011); consequently, new tools have been developed to aid organism identification. DNA barcoding (Hebert et al. 2003) is one of the most successful tools used to diagnose unknown specimens; however, the power of this tool heavily relies on the accuracy of its curated data.

The workflow leading to curated sequences starts with specimen sampling and preservation, followed by preliminary taxonomic identification (at higher ranks), molecular processing (digestion, amplification and sequencing) and finally the storage of any remaining voucher in appropriate collections. ‘Noise’ can be introduced in this sequence of tasks in a variety of ways, from specimen mislabeling to organism misidentification. The relevant outcome of such noise is that some of the sequences stored in public databases are associated with organism names to which they do not belong. Our main objective was to evaluate the amount of error for Hydrachnidia COI sequences in two of these databases, GenBank and BOLD.

We used two complementary approaches to identify errors in the sequences. First, we identified outlier sequences within clades on a reconstructed phylogenetic tree and compared them with those comprising other clades to determine the species to which the outlier most likely belongs. The phylogenetic tree was built on the assumption that sequences from the same species group together, that is, they have the same most recent common ancestor. Second, we assessed the extent to which sequences were equivalently identified as the same taxa in both databases by cross-referencing GenBank and BOLD sequences. For this last approach, one point to consider is that many of the sequences present in BOLD (especially those that have been published in a manuscript) are transferred to GenBank and vice versa (albeit in a smaller proportion). Therefore, there is a level of self-generated matching between databases; as such, most sequences that do not match are present only in GenBank.

Hydrachnidia is poorly represented in both databases: at the time of this study, GenBank had 4914 COI sequences, representing only 203 species from 56 (or 11%) of the presently known genera. In BOLD, where many sequences are private, there are sequences representing 37 families, 244 genera and 431 nominate species (species with an unspecific name—such as, e.g., *Eylais* sp.—were excluded from this account; assessed by 25 November 2020). In addition, very few species have more than one COI sequence in GenBank, suggesting that this gene is mainly used for species identification or phylogenetic inferences but not for population genetics, which requires a much higher number of sequences per species for robust analyses (e.g., Horreo and Fitze 2015).

DNA sequences with the same organism name potentially belong to different taxa, indicating that errors may have been produced by (1) incorrect species identification; (2) incorrect DNA electropherogram reading/interpretation (a usual source of errors in DNA analyses; Prieto et al. 2008); (3) DNA contamination; (4) sequence mislabeling; or (5) errors committed during the submission of sequences to databases. Although any of these are possible, we suspect that most, if not all, of the errors found in this study are primarily related with species identification, which is a challenging task in this type of organism and for this clade, especially to the species level (e.g., Stalstedt et al. 2013). Interestingly, sequence errors do not necessarily occur only when a high number of sequences is involved, as even species with only two available sequences (e.g., *Torrenticola amplexa*) present errors. Indeed, any number of sequences per species potentially contains a source of error. For instance, having the same species name associated with sequences that show >10% difference in similarity may be due to other causes besides misidentification. As we mentioned earlier and as B.P. Smith, author of some of the *Arrenurus* sequences listed in Table 4, commented “COI sequences can be shared occasionally whether by chance, hybridization or because of limited time since species divergence” (pers. comm., January 2020). In these cases, as in that of *Hydryphantes armentarius*/*H. parmulatus*, for which vouchers, images and publication are available (Valdecasas et al. 2019), a review of the taxonomic discordance, similar to the one conducted by Pentinsaari et al. (2020), may help resolve the underlying cause of these putative errors, thereby preventing future difficulties.

A drawback of the Hydrachnidia sequences available from public databases is that most are not identified to the species level (around 90%), and in those that are, errors caused by DNA contamination, species (mis)identification or DNA electropherogram reading/interpretation are present for 0.5% of the sequences, representing nearly 5% of the species and 11% of the genera for which molecular data exist. Although the proportion of sequences presenting errors is relatively small in Hydrachnidia, at least compared with other animal groups (e.g., in fishes, see Li et al. 2018), it could still be detrimental if these sequences are used in, for instance, systematic, phylogeographic or taxonomic studies. Their use could lead to erroneous phylogenetic trees, genetic variability estimations, phylogeographic inferences and species identification (e.g., when comparing sequences with BLAST), as well as flawed hypotheses and conclusions. Moreover, our comparison of sequences from GenBank and BOLD shows that the same sequence can be identified (or not) to the species level or can belong to a different species in the two databases. As also noted by others, improving the cross-referencing of sequences in these databases will, in general, increase their utility (Porter and Hajibabaei 2018).

However, some biologically relevant factors, and not human error, may be involved in some genetic misidentifications. Cryptic speciation (a process resulting in species that are morphologically identical but largely reproductively isolated) is known to occur widely in Acari (Scoracka et al. 2015), and may explain the paradoxical distribution of some taxa, for example, some non-parasitic water mites that seemingly have a wider distribution than parasitic ones (Yagui and Valdecasas 2020). Cryptic speciation is increasingly being studied in water mites (for recent literature and discussion, see e.g., Stalstedtet al. 2013; García-Jiménez et al. 2017; Pešić et al. 2017), which is contributing to the reestablishment of previously synonymized taxa. In the context presented here, taxonomic identification may be correct based on current taxonomic knowledge, but the phylogenetic analyses of DNA sequences could show incongruent relationships. Another process that should be considered for potentially erroneous molecular data is genetic introgression/hybridization. As mitochondrial information (mainly COI) appears to be predominantly used in molecular studies of Hydrachnidia, analyses that show differences in genetic and morphological identifications may be reflecting evidence of this process. For instance, molecular data could be identifying the maternal species of a hybrid that shares the morphology of the paternal species, leading to discordance between the two types of data (e.g., Pelaez et al. 2018). Incomplete lineage sorting could also affect the reliability of DNA barcoding initiatives and public DNA databases for species identification because the gene sequences used may not accurately reflect phylogenetic relationships among species (Linder and Rieseberg 2004). All of these factors must be taken into account when searching for potential errors in DNA databases.

In short, our current knowledge of the molecular characters of Hydrachnidia is very poor (the 203 barcoded species represent < 3% of the known species), despite the substantial number of new species discovered every year. Our case study also highlights the potential problems associated with relying on DNA sequences from public databases, particularly for species identification, and reinforces, once again, the need for improved controls and/or protocols to avoid intensifying errors in the genomics era. They also reveal the need for systematic taxonomic revisions for some Hydrachnidia clades: taxa that appear to be non-monophyletic may represent cases of cryptic diversity for which underlying mechanisms or processes need to be clarified, such as those related with cryptic species complexes, synonymization of taxa, hybridization, incomplete lineage sorting or sexual dimorphism (reviewed in Mutanen et al. 2016). Altogether, this situation leads to an underestimation of the true diversity of Hydrachnidia. Therefore, greater and accurate molecular data for the group are needed to support the maintenance of water mite biodiversity, particularly given the ever-increasing pressure being placed on freshwater ecosystems.

## Electronic Supplementary Material

Below is the link to the electronic supplementary material.

DNA alignment of the Hydrachnidia COI sequences used in this study.

Maximum Likelihood phylogenetic tree obtained from the analysis of Hydrachnidia COI sequences (Supplementary Material S1).

## Data Availability

All DNA sequences used in this study were obtained from GenBank and BOLD public databases. The COI sequence alignment and phylogenetic tree are provided as Supporting Information.

## References

[CR1] Alarcon-Elbal P, Garcia-Jimenez R, Pelaez ML, Horreo JL, Valdecasas AG (2020) Molecular correlation between larval, deutonymph and adult stages of the water mite *Arrenurus (Micruracarus) novus*. Life 10:108 32659940 10.3390/life10070108 PMC7400179

[CR2] Bickford D, Lohman DJ, Sodhi NS, Ng PKL, Meier R, Winker K, Ingram KK, Das I (2007) Cryptic species as a window on diversity and conservation. Trends Ecol Evol 22:148–155 17129636 10.1016/j.tree.2006.11.004

[CR4] Cook DR (1974) Water mite genera and subgenera. Mem Am Entomol Inst 21:1–860

[CR5] Ebach MC, Valdecasas AG, Wheeler QD (2011) Impediments to taxonomy and users of taxonomy: accessibility and impact evaluation. Cladistics 27:550–557 34875802 10.1111/j.1096-0031.2011.00348.x

[CR6] García-Jiménez R, Horreo JL, Valdecasas AG (2017) Minimal barcode distance between two water mite species from Madeira Island: a cautionary tale. Exp Appl Acarol 72:133–143. 10.1007/s10493-017-0147-5 28623498 10.1007/s10493-017-0147-5

[CR7] Glass DJ (2007) Experimental design for biologists. Cold Spring Harbor Laboratory Press, Cold Spring Harbor

[CR8] Guindon S, Dufayard JF, Lefort V, Anisimova M, Hordijk W, Gascuel O (2010) New algorithms and methods to estimate maximum-likelihood phylogenies: assessing the performance of PhyML 3. Syst Biol 59:307–321 20525638 10.1093/sysbio/syq010

[CR9] Harris DJ (2003) Can you bank on GenBank? Trends Ecol Evol 18:317–319

[CR10] Hebert PD, Cywinska A, Ball SL, deWaard JR (2003) Biological identifications through DNA barcodes. Proc Nat Acad Sci USA 270:313–321 10.1098/rspb.2002.2218 PMC1691236 12614582

[CR11] Horreo JL (2012) ’Representative genes’, is it OK to use a small amount of data to obtain a phylogeny that is at least close to the true tree? J Evol BIol 25:2661–2664 22998749 10.1111/j.1420-9101.2012.02622.x

[CR12] Horreo JL, Fitze PS (2015) Population structure of three *Psammodromus* species in the Iberian Peninsula. PeerJ 3:e994 26056622 10.7717/peerj.994 PMC4458133

[CR13] Janssen T, Karssen G, Couyreur M, Waeyenberge L, Bert W (2017) The pitfalls of molecular species identification: a case study within the genus *Pratylenchus* (Nematoda: Pratylenchidae). Nematology 10:1179–1199

[CR15] Li X, Shen X, Chen X, Xiang D, Murphy RW, Shen Y (2018) Detection of potential problematic Cytb gene sequences of fishes in GenBank. Front Genet 3:30 10.3389/fgene.2018.00030 PMC5808227 29467794

[CR16] Linder CR, Rieseberg LH (2004) Reconstructing patterns of reticulate evolution in plants. Am J Bot 91:1700–1708 18677414 PMC2493047

[CR17] Lis JA, Lis B, Ziaja DJ (2016) In BOLD we trust? A commentary on the reliability of specimen identification for DNA barcoding: a case study on burrower bugs (Hemiptera: Heteroptera: Cydnidae). Zootaxa 4114:83–86 27395114 10.11646/zootaxa.4114.1.6

[CR18] Meyer CP, Paulay G (2005) DNA barcoding: error rates based on comprehensive sampling. PLoS Biol 3:e422 16336051 10.1371/journal.pbio.0030422 PMC1287506

[CR19] Minh BQ, Nguyen MAT, von Haeseler A (2013) Ultrafast approximation for phylogenetic bootstrap. Mol Biol Evol 30:1188–1195 23418397 10.1093/molbev/mst024 PMC3670741

[CR20] Mutanen M, Kivelä SM, Vos RA, Doorenweerd C, Ratnasingham S, Hausmann A, Huemer P, Dinca V, van Nieukerken EJ, Lopez-Vaamonde C, Vila R, Aarvik L, Decaëns T, Efetov KA, Hebert PDN, Johnsen A, Jarsholt O, Pentinsaari M, Rougerie R, Segerer A, Tarmann G, Zahiri R, Godfray HCJ (2016) Species-level para-and polyphyly in DNA barcode gene trees: strong operational bias in European Lepidoptera. Syst Biol 65:1024–1040 27288478 10.1093/sysbio/syw044 PMC5066064

[CR21] Nguyen L-T, Schmidt HA, von Haeseler A, Minh BQ (2015) IQ-TREE: a fast and effective stochastic algorithm for estimating maximum likelihood phylogenies. Mol Biol Evol 32:268–374 25371430 10.1093/molbev/msu300 PMC4271533

[CR22] Page TJ, Satish C, Hughes JM (2005) The taxonomic feedback loop: symbiosis of morphology and molecules. Biol Lett 1:139–142 17148149 10.1098/rsbl.2005.0298 PMC1626211

[CR23] Pentinsaari M, Ratnasingham S, Miller SE, Hebert PDN (2020) BOLD and GenBank revisited—do identification errors arise in the lab or in the sequence libraries? PLoS ONE 15(4):e0231814. 10.1371/journal.pone.0231814 32298363 10.1371/journal.pone.0231814 PMC7162515

[CR24] Pelaez ML, Valdecasas AG, Martinez D, Horreo JL (2018) Towards unravelling of the slug *A. ater-A. rufus* complex (Gastropoda Arionidae): new genetic approaches. Web Ecol 18:115–119

[CR25] Pešić V, Asadi M, Cimpean M, Dabert M, Esen Y, Gerecke R, Martin P, Savic A, Smit H, Stur E (2017) Six species in one: evidence of cryptic speciation in the *Hygrobates fluviatilis* complex (Acariformes, Hydrachnidia, Hygrobatidae). Syst Appl Acarol 22(9):1327–1377

[CR26] Porter TM, Hajibabaei M (2018) Over 2.5 million COI sequences in GenBank and growing. PLoS ONE 13:e0200177 30192752 10.1371/journal.pone.0200177 PMC6128447

[CR27] Prieto L, Alonso A, ALves C, Crespillo M, Montesino M, Picornell A, Brehm A, Ramírez JL, Whittle MR, Anjos MJ, Boschi I, Buj J, Cerezo M, Cardoso S, Cicarelli R, Comas D, Corach D, Doutremepuich C, Espinheira RM, Fernández-Fernández I, Filippini S, Garcia-Hirscgfeld J, González A, Heinrichs B, Lorente JA, Mechoso B, Nacarro I, Pagano S, Pestano JJ, Puente J, Vidal-Rioja L, Vullo C, Salas A (2008) 2006 GEP-ISFG collaborative exercise on mtDNA: reflections about interpretation, artefacts, and DNA mixtures. For Sci Int 2:126–133 10.1016/j.fsigen.2007.10.010 19083807

[CR28] Proctor HC, Smith IM, Cook DR, Smith BP (2015) Subphylum chelicerata, class arachnida. In: Thorp and Covich's Freshwater Invertebrates, Academic Press, London

[CR30] Scoracka A, Magalhaes S, Rector BG, Kuczynski L (2015) Cryptic speciation in the Acari: a function of species lifestyles or our ability to separate species? Exp Appl Acarol 67:165–182 26209969 10.1007/s10493-015-9954-8 PMC4559570

[CR31] Stalstedt J, Bergsten J, Ronquist F (2013) “Forms” of water mites (Acari: Hydrachnidia): intraspecific variation or valid species? Ecol Evol 3:3415–3435 24223279 10.1002/ece3.704 PMC3797488

[CR32] Valdecasas AG, Garcia-Gimenez R, Marin F (2019) Sobre la presencia de dos especies raras de ácaros acuáticos (Parasitengona, Hydrachnidia) en la Península Ibérica. Rev Iber Aracnol 35:33–37

[CR34] Wagner TC, Bodem J (2017) Sequence errors in foamy virus sequences in the GenBank database: resequencing of the prototypic foamy virus proviral plasmids. Archiv Virol 162:1141–1144 10.1007/s00705-016-3206-z 28040837

[CR33] Yagui H, Valdecasas AG (2020) Does parasitism mediate water mite biogeography? Syst Appl Acarol 25:1552–1560

