# Correction: An evaluation of errors in the mitochondrial COI sequences of Hydrachnidia (Acari, Parasitengona) in public databases

**DOI:** 10.1007/s10493-024-00979-4

**Published:** 2024-11-27

**Authors:** María L. Peláez, José L. Horreo, Ricardo García-Jiménez, Antonio G. Valdecasas

**Affiliations:** 1 Museo Nacional de Ciencias Naturales, C/José Gutiérrez Abascal, 2, 28006 Madrid, Spain; 2 UMIB Research Unit of Biodiversity (UO, CSIC, PA), Oviedo University - Campus Mieres, C/Gonzalo Gutiérrez Quirós s/n, 33600 Mieres, Spain; 3 Department of Genetics, Physiology and Microbiology, Complutense University of Madrid, C/Jose Antonio Novais 12, 28040 Madrid, Spain

**Experimental and Applied Acarology (2022) 86:371–384**

10.1007/s10493-022-00703-0

The article “An evaluation of errors in the mitochondrial COI sequences of Hydrachnidia (Acari, Parasitengona) in public databases“, written by María L. Peláez et al. was originally published Online First without Open Access. After publication in volume 86, issue 3, page 371–384 the author decided to opt for Open Choice and to make the article an Open Access publication. Therefore, the copyright of the article has been changed to © The Author(s) 2022 and the article is forthwith distributed under the terms of the Creative Commons Attribution 4.0 International License, which permits use, sharing, adaptation, distribution and reproduction in any medium or format, as long as you give appropriate credit to the original author(s) and the source, provide a link to the Creative Commons licence, and indicate if changes were made. The images or other third party material in this article are included in the article’s Creative Commons licence, unless indicated otherwise in a credit line to the material. If material is not included in the article’s Creative Commons licence and your intended use is not permitted by statutory regulation or exceeds the permitted use, you will need to obtain permission directly from the copyright holder. To view a copy of this licence, visit http://creativecommons.org/licenses/by/4.0/.

